# Evaluation of Fosphenytoin Therapeutic Drug Monitoring in the Neurocritical Care Unit

**DOI:** 10.1007/s40268-019-00292-1

**Published:** 2020-01-10

**Authors:** Mandee Noval, Hyunuk Seung, Michael Armahizer

**Affiliations:** 1 University of Maryland Medical Center, 22 S. Greene Street, Baltimore, MD USA; 2 University of Maryland School of Pharmacy, 20 N Pine St, Baltimore, MD USA

## Abstract

**Objective:**

The aim of this study was to determine whether the current method of calculating a fosphenytoin reloading dose results in a therapeutic free phenytoin level on subsequent days.

**Methods:**

Medical records of patients receiving fosphenytoin in the neurocritical care unit between July 2017 and June 2018 were screened. Included patients were those who had received at least three doses of fosphenytoin and required reloading doses according to concentrations obtained through therapeutic drug monitoring. Free phenytoin levels were categorized based on the prespecified patient-specific target range, generally between 1.5 and 2.5 mcg/mL.

**Results:**

Of the fosphenytoin reloading doses administered, 48% (73/152) resulted in a therapeutic free phenytoin concentration on the subsequent day, with the remaining 52% resulting in nontherapeutic levels (39% subtherapeutic, 13% supratherapeutic). Our evaluation of reloading dose calculation strategies indicated that patients were two times as likely to obtain a therapeutic level when a modified pharmacokinetic equation omitting the use of volume of distribution or salt formulation was used (58%, *n* = 39) than they were with doses calculated using the current pharmacokinetic model (41%, *n* = 20) or doses based on provider preference (39%, *n* = 14).

**Conclusion:**

The current method of calculating a fosphenytoin reloading dose in the critically ill population does not consistently result in therapeutic concentrations. With multiple factors affecting the pharmacokinetics of critically ill patients, the creation of a new pharmacokinetic model with less emphasis on volume of distribution may more consistently result in therapeutic concentrations.

## Key Points

**Table:** 

Fosphenytoin pharmacokinetics remain unpredictable, especially when the drug is used in critically ill patients with fluctuating factors affecting drug levels and often necessitating dose adjustments.
Current methods of calculating a reloading dose in patients with subtherapeutic free phenytoin concentration levels do not consistently result in therapeutic free concentrations on subsequent days.
A modified calculation with less emphasis on volume of distribution was associated with a twofold increased chance of attaining a therapeutic free phenytoin level on the subsequent day.

## Background

Phenytoin and the prodrug fosphenytoin are frequently used in the treatment of critically ill patients experiencing seizures or as prophylaxis in neurologically injured patients [1–3]. Phenytoin exhibits nonlinear elimination pharmacokinetics and has been known to have high interpatient dosing variability, with therapeutic drug monitoring being the standard of care in patients receiving either agent [4–6]. The volume of distribution (*V*_d_) of phenytoin is estimated as approximately 0.7–1.0 L/kg but may be routinely unpredictable as it increases with both dose and rate, resulting in heightened difficulty when trying to use pharmacokinetic models to predict drug concentrations [3, 7, 8]. In addition, approximately 90% of phenytoin is bound to plasma albumin, with drug levels being highly affected by conditions or medications that alter protein binding [3, 9].

While phenytoin itself has unique pharmacokinetic properties, so does the patient population where it's used most often, including many critically ill patients requiring rapid administration for seizure control. Several of the previously mentioned factors are increasingly prevalent in this population, including the presence of hypoalbuminemia affecting protein binding, hepatic dysfunction, renal dysfunction, or concomitant drug interactions [7–9]. These factors, along with the increased number of complex disease states or comorbidities that may alter volume status and drug distribution, further highlight the importance of therapeutic drug monitoring [4]. Critical illness also has the potential to affect overall hepatic metabolism of the drug, adding to the difficulty of obtaining a therapeutic free concentration and resulting in the need for frequent dose adjustments based on therapeutic drug monitoring [7, 8, 10].

Initial dosing strategies for phenytoin consist of administering an intravenous loading dose of phenytoin or fosphenytoin between 10 and 20 mg/kg of phenytoin equivalents followed by initiation of a maintenance regimen [3, 6, 11, 12]. Free and/or total levels are then measured approximately 48–72 h after therapy initiation, with free levels in the range of 1.0–2.5 mcg/mL often considered to be therapeutic, although a patient’s clinical status must also be considered to determine treatment goal and presumed therapeutic effect [6]. If free levels remain low after therapies are initiated, a reloading dose may be administered to increase the likelihood of attaining a therapeutic level. Pharmacokinetic equations can be used to calculate a reloading dose according to the desired goal concentration; however, the use of these equations has not been validated in critically ill populations. The current method of calculating a reloading dose incorporates both the current and the target free level (mcg/mL), along with the estimated V_d_ of the drug (0.7 L/kg), the patient’s actual body weight (kg), and the salt formulation (0.92) (Eq. 1).

1 $${\text{Current Equation Calculated Dose}} = \frac{{\left[ {\left( {{\text{Target Free PHT}} - {\text{Current Free PHT}}} \right) \times 10} \right] \times 0.7 \times {\text{weight}}}}{0.92}$$

where PHT is phenytoin.

Although this equation is commonly used in practice, we hypothesized that its use does not consistently result in therapeutic concentrations in critically ill patients because of their varied pharmacokinetic parameters and the unpredictable *V*_d_ of phenytoin.

The purpose of this study was to determine whether the current method of calculating a fosphenytoin reloading dose routinely results in a therapeutic free phenytoin level in the critically ill population. The primary outcome was to determine the overall incidence of patients achieving therapeutic concentrations on the subsequent day. Secondary outcomes included the development of a new pharmacokinetic dosing strategy to achieve therapeutic free levels and to evaluate the incidence and effect of concomitant drug interactions in patients receiving fosphenytoin.

## Methods

This retrospective chart review evaluated patients receiving fosphenytoin in the neurocritical care unit (NCCU) at the University of Maryland Medical Center (UMMC) between 1 July 2017 and 30 June 2018. Patients were included in the study if they were aged 18–80 years, had received at least 3 days of fosphenytoin therapy with at least one reloading dose of intravenous fosphenytoin or phenytoin, and had both pre- and post-reloading dose free phenytoin levels collected. Exclusion criteria included pregnant patients, patients who did not require reloading doses, and patients receiving oral phenytoin exclusively. This study was approved by the Institutional Review Board at UMMC.

Demographic information was collected for each patient, including age, sex, height, and weight. Laboratory data at the day of reloading dose administration was collected, including serum creatinine, serum albumin, aspartate aminotransferase, alanine aminotransferase, and total bilirubin. If laboratory results were not available for the day of fosphenytoin reloading dose administration, the most recent laboratory data available during index admission were collected. Pertinent past medical history was obtained through chart review of confirmed diagnoses, including the presence of renal dysfunction, hepatic dysfunction, or disease states known to impact *V*_d_ such as heart failure. Renal dysfunction, particularly the presence of acute kidney injury, was defined using RIFLE criteria [13, 14]. Hepatic dysfunction was defined as a documented history of cirrhosis or the presence of elevated transaminases (≥ 3 × upper limit of normal) reflecting the potential for acute liver injury. Concomitant medications with the potential to interact with fosphenytoin were recorded, including aspirin, carbamazepine, dexamethasone, methotrexate, phenobarbital, theophylline, and valproic acid. The medication administration record during the index admission was manually reviewed for receipt of the specified medications. These drugs have been shown to alter phenytoin concentrations by competing for protein binding sites or through cytochrome P450 2C9/2C19 interactions and were chosen based on similar studies.

Fosphenytoin indication and goal free level were obtained through chart review of NCCU provider notes. The definition of a therapeutic free phenytoin level was based on documented goal levels, ranging from 1 to 2.5 mcg/mL depending on patient- or provider-specific goals. If a goal level was undocumented, we assumed a goal of 1.5–2.5 mcg/mL according to institution practice. Phenytoin samples were run using the VITROS^®^ 6000 Integrated System from Ortho Clinical Diagnostics. Free phenytoin levels were most often collected with morning laboratory tests, approximately 2–4 h before administration of the morning fosphenytoin dose. Total phenytoin levels were drawn on occasion, at provider discretion, typically upon admission if transferred from an outside hospital.

Determination of reloading dose strategy was identified retrospectively by comparing the administered dose and the calculated dose using the current reloading dose equation (Eq. 1) or a modified equation eliminating the use of *V*_d_ and salt formulation (Eq. 2).

2 $${\text{Modified Equation Calculated Dose}} = \left[ {\left( {{\text{Target Free PHT}} - {\text{Current Free PHT}}} \right) \times 10} \right] \times {\text{weight}}$$

Doses were considered to be calculated using either equation if they were within 250 mg of the predicted value as dose rounding is common practice at our institution. Doses that were not within 250 mg of the predicted value using either equation were considered to not be based on an equation.

Descriptive statistics were used to report frequencies and ranges. Normally distributed data are reported as means ± standard deviations. Bivariate analysis was performed to measure associations between all predictors and outcomes. For bivariate analysis, a logistic regression model was used for outcomes with independent factors. Odds ratios with 95% confidence intervals were calculated to measure associations between outcomes and effects, with a *p* value < 0.05 considered statistically significant. Data analysis was performed using SAS version 9.4 (SAS Institute, Cary, NC, USA).

## Results

A total of 155 patients were reviewed, with 51 patients meeting the inclusion criteria. From these 51 patients, 152 reloading doses of intravenous phenytoin or fosphenytoin were analyzed. The study population was mostly female (60.8%) with a mean age of 59 ± 14.4 years, a mean weight of 85.4 ± 22.2 kg, and a mean body mass index (BMI) of 30.1 ± 7.2 kg/m^2^ (Table 1). The most common admission diagnoses for those in the NCCU receiving fosphenytoin were status epilepticus (54.9%), hemorrhagic stroke (29.4%), and ischemic stroke (2.0%). Renal dysfunction was highly prevalent, with 33 patients (64.7%) experiencing some degree of decline in renal function, whereas hepatic dysfunction was less apparent, with only five patients (9.8%) experiencing abnormalities in liver function. Other comorbidities assessed included the presence of a preexisting seizure disorder (25.5%) or heart failure (5.9%).

**Table 1:** Baseline characteristics

Characteristics	Patients (*n* = 51)
Age (years)	59.2 ± 14.4
Female	31 (60.8)
Weight (kg)	85.4 ± 22.2
BMI (kg/m^2^)	30.1 ± 7.2
Admission diagnosis	
Status epilepticus	28 (54.9)
Subarachnoid hemorrhage	9 (17.6)
Intracerebral hemorrhage	6 (11.8)
Acute ischemic stroke	1 (2.0)
Other	7 (13.7)
Comorbidities	
Overall renal dysfunction	33 (64.7)
Acute kidney injury	21 (41.1)
Chronic kidney disease	12 (23.5)
Continuous renal replacement therapy	3 (5.9)
Hemodialysis	1 (2.0)
Preexisting seizure disorder	13 (25.5)
Overall hepatic dysfunction	5 (9.8)
Heart failure	3 (5.9)

Data are presented as mean ± standard deviation or *N* (%) unless otherwise indicated

*BMI* body mass index

Fosphenytoin was most often prescribed for treatment of seizures (92.2%) compared with prophylaxis (9.8%) (Table 2). Free phenytoin targets varied according to patient and provider, with most patients having a target free phenytoin goal of 1.5–2.0 mcg/mL (25.5%) or an unspecified patient-specific goal (31.4%), assumed to be 1.5–2.5 mcg/mL at our institution. The average initial loading dose administered was approximately 18 mg/kg, though loading doses were not consistently reported in patients transferred from outside institutions.

**Table 2:** Fosphenytoin dosing information

Dosing	Patients
Fosphenytoin indication (*n* = 51)	
Seizures	47 (92.2)
Home medication	5 (9.8)
Prophylaxis	4 (7.8)
Free phenytoin target goal (*n* = 51)	
1.0–2.0 mcg/mL	4 (7.8)
1.5–2.0 mcg/mL	13 (25.5)
1.5–2.5 mcg/mL	11 (21.6)
2.0–2.5 mcg/mL	7 (13.7)
Unspecified	16 (31.4)
Initial loading dose (mg/kg)	18.2 ± 4.4
Reloading dose calculation strategy (*n* = 152)	
Equation	49 (32.2)
Equation without *V*_d_ or salt formulation	67 (44.1)
Provider preference	36 (23.7)

Data are presented as mean ± standard deviation or N (%) unless otherwise indicated

*V*_*d*_ volume of distribution

Of fosphenytoin reloading doses that were administered, 48% (73/152) of doses resulted in a therapeutic free phenytoin concentration on the subsequent day, with the remaining 52% resulting in nontherapeutic levels (39% subtherapeutic, 13% supratherapeutic) (Table 3). Total levels were rarely collected (9/152) and were not frequently used to calculate reloading doses. When evaluating administered doses to determine whether they were based on calculations, we identified differences in the ability to obtain therapeutic free phenytoin levels. Among doses administered, 67 (44%) were based on a calculation not using *V*_d_ or salt formulation, 49 (32%) were based on a calculation using *V*_d_ and salt formulation, and 36 (24%) were not based on a calculation. Doses calculated without using *V*_d_ or salt formulation resulted in the highest percentage of therapeutic free phenytoin concentrations (58%, *n* = 39) compared with doses calculated using V_d_ and salt formulation (41%, *n* = 20) and doses not based on a calculation (39%, *n* = 14).

**Table 3:** Classification of fosphenytoin free levels stratified by dosing strategy

Free phenytoin levels	Within goal	Below goal	Above goal
All doses (*n* = 152)	73 (48.0)	59 (38.8)	20 (13.2)
Dosing strategy used			
Equation (*n* = 49)	20 (40.8)	28 (57.1)	1 (2.1)
Equation without *V*_d_/SF (*n* = 67)	39 (58.2)	18 (26.9)	10 (14.9)
Provider preference (*n* = 36)	14 (38.9)	13 (36.1)	9 (25.0)

Data are presented as *n* (%)

*SF* salt formulation, *V*_*d*_ volume of distribution

Using a bivariate analysis, we identified that the use of a modified pharmacokinetic equation without the addition of *V*_d_ and salt formulation was associated with a twofold increase in achieving therapeutic levels (odds ratio 2.10; 95% confidence interval 1.08–4.01) (Table 4). Other factors assessed, including the presence of organ dysfunction and drug interactions, were not statistically significantly associated with the incidence of attaining therapeutic levels.

**Table 4:** Bivariate analysis of factors associated with attainment of therapeutic free levels

Variable	Odds ratio (95% CI)	*P* value
Equation dosing	0.65 (0.32–1.30)	0.22
Equation without *V*_d_ + SF	2.10 (1.08–4.01)	0.03
Provider dosing	0.62 (0.28–1.32)	0.21
BMI ≥ 30	0.85 (0.44–1.61)	0.61
Renal dysfunction	0.57 (0.28–1.16)	0.12
Liver dysfunction	0.71 (0.19–2.61)	0.60
Concomitant ASA	0.75 (0.94–4.70)	0.07
Concomitant dexamethasone	0.84 (0.36–1.94)	0.68
Concomitant phenobarbital	0.77 (0.37–1.48)	0.43
Concomitant VPA	1.09 (0.38–3.08)	0.87

*ASA* acetylsalicylic acid (aspirin), *BMI* body mass index, *CI* confidence interval, *SF* salt formulation, *V*_*d*_ volume of distribution, *VPA* valproic acid

The overall incidence of drug interactions in the study population was relatively high, with 26 (51%) patients having at least one concomitant medication that may interfere with phenytoin levels and five (9.8%) patients experiencing more than one drug interaction (Table 5). Aspirin was the drug most commonly involved interaction (27.5%, *n* = 14) with the potential to displace phenytoin from binding sites, although this is increasingly rare at the prophylactic doses often used, followed by dexamethasone (13.7%, *n* = 7) and phenobarbital (13.7%, *n* = 7).

**Table 5:** Incidence of drug interactions

Drug	Patients (*n* = 51)
Overall incidence of drug interactions	26 (51.0)
Aspirin	14 (27.5)
Carbamazepine	0 (0.0)
Dexamethasone	7 (13.7)
Methotrexate	0 (0.0)
Phenobarbital	7 (13.7)
Theophylline	0 (0.0)
Valproic acid	3 (5.9)

Data are presented as *n* (%)

## Discussion

The pharmacokinetics of phenytoin are known to be unpredictable, and therapeutic drug monitoring has been well-established as a method of determining drug efficacy and toxicity. With numerous factors affecting drug distribution and the potential for drug saturation, dosing needs remain highly individualized throughout treatment courses. Previous studies have evaluated phenytoin therapeutic drug monitoring in various populations, including obesity and critical illness; however, few studies have evaluated the accuracy of dose adjustments in these patients after initial therapy [8, 11].

Overall, we evaluated three different strategies for calculating a reloading dose to determine whether a difference existed in the incidence of obtaining subsequent therapeutic free phenytoin concentrations. Our analysis of 51 patients receiving 152 doses showed that patients had the greatest chance of achieving a therapeutic level when the modified equation omitting *V*_d_ and salt formulation was used. The use of the current method of calculating a reloading dose more commonly resulted in subtherapeutic concentrations, showing that it is more likely to underdose patients. The *V*_d_ used in the current Eq. (0.7 L/kg) is an estimate of the predicted value; however, previous studies have shown that the V_d_ of phenytoin is affected by fluid status, weight, and albumin, all of which fluctuate significantly in critically ill patients, making it difficult to predict a standard *V*_d_ applicable for all [9]. Though *V*_d_ is likely the pharmacokinetic parameter with the lowest interindividual variability, the value that should be used in this increasingly complex population remains uncertain and begs the question of whether *V*_d_ should be increased to a value of 1 L/kg to better represent the difference in critically ill patient pharmacokinetics, essentially omitting it from the equation.

When using a bivariate analysis to determine whether any factors were associated with an increased incidence of attaining a therapeutic level, patients receiving doses based on the modified equation were two times as likely to achieve a therapeutic level. Interestingly, a large number of patients had renal dysfunction, which is thought to be associated with an increased fraction of free phenytoin, expected to correlate with an increase in *V*_d_ and the potential need for decreased doses [9]. Our bivariate analysis found no statistically significant association between phenytoin use, renal dysfunction, and obtaining therapeutic drug concentrations, and these patients were just as likely to experience subtherapeutic concentrations. Other factors analyzed, including the use of interacting medications, also showed no association, although these medications are known to have the potential to affect drug concentrations, and providers should still be cognizant of concomitant use.

Many patients (51%) experienced at least one drug interaction, with most involving concomitant use of a medication with the potential to displace phenytoin from albumin binding sites (aspirin, valproic acid). This would, in theory, result in falsely elevated total drug concentrations, whereas the unbound concentration may remain unchanged or potentially increase if the displacing drug also inhibits phenytoin metabolism. It is possible that the true effect of such interactions were not seen because a low dose of aspirin was used in all patients and phenytoin itself is an inducer, potentially cancelling out the effects of other interactions. This effect also may not have been as apparent since free levels were primarily collected as opposed to total, with most patients still experiencing subtherapeutic concentrations.

Our study has some limitations. First, it was retrospective in design, making it difficult to ensure free levels collected were “true” troughs. At our institution, free phenytoin levels are analyzed twice daily, with levels most often obtained with morning laboratory tests for convenience. Although this may affect study generalizability, this is the standard method of dose adjustment at our institution and mimics practicality. In addition, the dosing strategy used was extrapolated from predicted calculations, but we cannot tell whether the dosing strategy was deliberately chosen by the ordering provider or whether assistance was provided by a clinical pharmacist. Also, the list of drug interactions and comorbidities is not all inclusive but selected based on commonly included medications throughout the literature. Finally, because of the retrospective design, only a small number of patients had certain comorbidities of interest, including hepatic dysfunction, limiting our ability to make conclusions regarding the effects these comorbidities exerted on phenytoin levels.

## Conclusion

The current method of calculating a fosphenytoin reloading dose in the critically ill population does not consistently result in therapeutic levels, leading to more patients with persistently subtherapeutic phenytoin concentrations. With multiple factors affecting pharmacokinetics in critically ill patients, the use of a modified pharmacokinetic equation with less emphasis on *V*_d_ may result in therapeutic concentrations more consistently than current strategies.
